# Effects of climate warming on plant autotoxicity in forest evolution: a case simulation analysis for *Picea schrenkiana* regeneration

**DOI:** 10.1002/ece3.2315

**Published:** 2016-07-23

**Authors:** Xiao Ruan, Cun‐De Pan, Run Liu, Zhao‐Hui Li, Shu‐Ling LI, De‐An Jiang, Jing‐Chi Zhang, Geoff Wang, Yin‐Xian Zhao, Qiang Wang

**Affiliations:** ^1^ Ningbo Institute of TechnologyZhejiang UniversityNingbo315100China; ^2^ College of Forestry and HorticultureXinjiang Agricultural UniversityÜrümqi830052China; ^3^ College of Life scienceZhejiang UniversityHangzhou310058China; ^4^ College of Forest Resources and EnvironmentNanjing Forest UniversityNanjing210037China; ^5^ School of AgriculturalForest and Environmental SciencesClemson UniversityClemson29631South Carolina

**Keywords:** Climate warming, DHAP, *Picea schrenkiana*, plant autotoxicity, seed germination, seedling growth

## Abstract

In order to explore how plant autotoxicity changes with climate warming, the autotoxicity of *P. schrenkiana* needles' water extract, organic extract fractions, and key allelochemical DHAP was systemically investigated at the temperature rising 2 and 4°C based on the data‐monitored soil temperature during the last decade in the stage of Schrenk spruce regeneration (seed germination and seedling growth). The results showed that the criterion day and night temperatures were 12°C and 4°C for seed germination, and 14°C and 6°C for seedling growth, respectively. In the presence of water extract, the temperature rise of 2°C significantly inhibited the germination vigor and rate of *P. Schrenkiana* seed, and a temperature rise of 4°C significantly increased the inhibition to the seedling growth (*P *<* *0.05). Among the three organic fractions, the low‐polar fraction showed to be more phytotoxic than the other two fractions, causing significant inhibitory effects on the seed germination and growth even at low concentration of 0.1 mg/mL, and the inhibition effect was enhanced as temperature increased. The temperature rise significantly enhanced the promotion effect of DHAP, while the inhibition effect of temperature rise became less important with increasing concentration of DHAP. This investigation revealed that autotoxicity of *P. schrenkiana* was affected by the climate warming. As expected, it provided an insight into the mechanism and effectiveness of allelopathy in bridging the causal relationship between forest evolution and climate warming.

## Introduction

The phenomenon of one plant's growth inhibited by chemicals released from another plant into environment is generally defined as allelopathy (Callaway and Vivanco 2006). The term “allelopathy” was first used by Hans Molisch from a physiological perspective to describe the effect of ethylene on fruit ripening (Duke 2010). Allelochemicals, delivered through decomposition, volatilization, leaching, and root exudation (El Mehdawi et al. 2011), play an important role in the distribution of plant populations (Wardle et al. 2011), the succession of communities, as well as the nutrient chelation (Vanderstukken et al. 2014), and were also suggested as a mechanism driving exotic plant invasion (Mangla and Callaway 2008). In spite of usually being interspecific, allelopathy may also occur within the same species the so‐called autotoxicity. Up to now, autotoxicity has been documented in a number of coniferous species and believed to be involved in natural and managed ecosystems (Fernandez et al. 2008).

Although it is still under debate whether and how allelopathy drive forest succession, ecosystem‐level alleopathic effect has been argued as a cause for regeneration failure of conifer, evidenced by examples from sitka spruce, scots pine, norway spruce, black spruce, red pine, jack pine, balsam fir, douglas fir, western hemlock, western red cedar, and amabilis fir (Mallik 2003). After analyzing the data from more than 20 years of fieldwork on six major tree species (*Cryptocarya chinensis*,* Cryptocarya concinna*,* Schima superba*,* Castanopsis chinesis*,* Caryota ochlandra*, and *Castanopsis fissa*) in Dinghu Mountain, Peng et al. (2004) found that all these species contained allelochemicals affecting their succession and then hypothesized that allelopathy together with light and water may be the major driving forces in tropical and subtropical forest succession and evolution.

Climatic changes along with other factors to influence ecological environment will undoubtedly affect the recruitment of plants and population dynamics subsequently (Dalgleish et al. 2010). Early stages of plant growth are expected to be more sensitive to climate change than adult stages and, as such, represent a major bottleneck to recruitment (Donohue et al. 2010). Seedling emergence is usually synchronized with seasonal changes in the environment (Fenner and Thompson 2005). Germination of some species happens soon after dispersal, whereas that of other dormant species is postponed until a favorable season when seedlings are suitable to survive, grow, and go on to reproduce (Walck et al. 2011). Owing to the influence on the evolution of postgermination traits and the fitness, germination experiences strong selection, and phenotypic plasticity may be subject to strong selection as well (Hoffman et al. 2010). Thus, there is the pertinent issue whether adaptation of seed and seedling traits can track the speed of climate change (Hovenden et al. 2008). Moreover, such adaptation of annual (or herbaceous) species having rapid life cycles may go faster than that of perennial (or woody) species (Ibanez et al. 2007). Current understanding of the evolutionary responses influencing plant regeneration is basically obtained from the observations on weedy species (Potvin and Tousignant 1996). At least for some plants, it appears that seed traits could evolve relatively fast to catch up with climate change (Sherry et al. 2007). For the plasticity/adaptations of tree species, however, we know very little about their germination behavior in response to environmental changes (Hedhly et al. 2009; Logant et al. 2013).

As one of the most important zonal vegetations, Schrenk spruce (*Picea schrenkiana Fisch*. et Mey.) is an endemic species in Middle Asia and the mountains of Asia. In China, Schrenk spruce is mainly distributed on the northern and southern slopes of Tianshan Mountains, and the northern slope of Kunlun Mountain West. As widely documented, however, the natural regeneration of Schrenk spruce has been in jeopardy (Wang et al. 2006). Previous investigation showed that all the water extract of *P. Schrenkiana* needles and its organic extract fractions of diethyl ether, ethyl acetate, and *n*‐butanol all exhibited autotoxic effects on seed germination and seedling growth. Analysis for the chemical composition of *P. Schrenkiana* needles revealed a great number of secondary metabolites that might act as allelochemicals (Li et al. 2011). The phenolic compound 3,4‐dihydroxy‐acetophenone (DHAP) isolated by bioassay‐guided fractionation from the diethyl ether fraction was proved to be the major autotoxic chemical in *P. Schrenkiana* needles (Ruan et al. 2011).

In this study, we take the analysis for *P. schrenkiana* regeneration as a case. A series of experiments were designed and conducted to discuss the effect of climate warming on plant autotoxicity and provided a means for the recruitment of plants and population dynamics affected by ecological environment.

## Materials and Methods

### Geography and climate of the surveyed field

As one of the largest mountain systems in Central Asia, Tianshan Mountain Range covers 800,000 km^2^ between 69°–95°E and 39°–46°N and stretches approximately 2500 km from the southwest to northeast in one of the most arid midlatitude zones on Earth. The forest zone of the Tianshan Mountain is limited to altitudes between 1500 and 2700 m where the climate is sufficiently wet and warm. The soil is classified as brown forest soil covered with a thick humus layer. Annually, the mean temperature, precipitation, aporation, and relative humidity are 2°C, 400–600 mm, 980–1150 mm, and 65%, respectively, while the aridity index is 1.4 and the frost‐free period is 89 days.

The thermal tree line lies at 2700 m where the average temperature in the warmest month is about 10°C, and the areas below 1500 m are generally too dry to support boreal forest. The multilayer structure of forest contains different types of plants, including trees, shrubs, ferns, grasses, and moss. *P. schrenkiana* mixed with *Larix sibiricain* in the eastern region are the major forest type of Tianshan Mountains. The dominant species of understory shrubs are *Juniperus pseudosabina* and *Juniperus sabina*. *Dicranum scoparium* and *Hepnum revolutumare* represent the main species of understory bryophytes, and the herbs mainly include *Stellaria songorica* and *Cortusa brother* (Wang et al. 2004). Field observations at different altitudes in Tianshan Mountains indicated that the rush period of the spruce seed germination appeared in early May to mid June, the critical period of the sprout growth appeared in late June to late July, and the plants grew very slowly after August due to more rain and snow.

### Field monitoring of topsoil temperature

**Figure 1:**
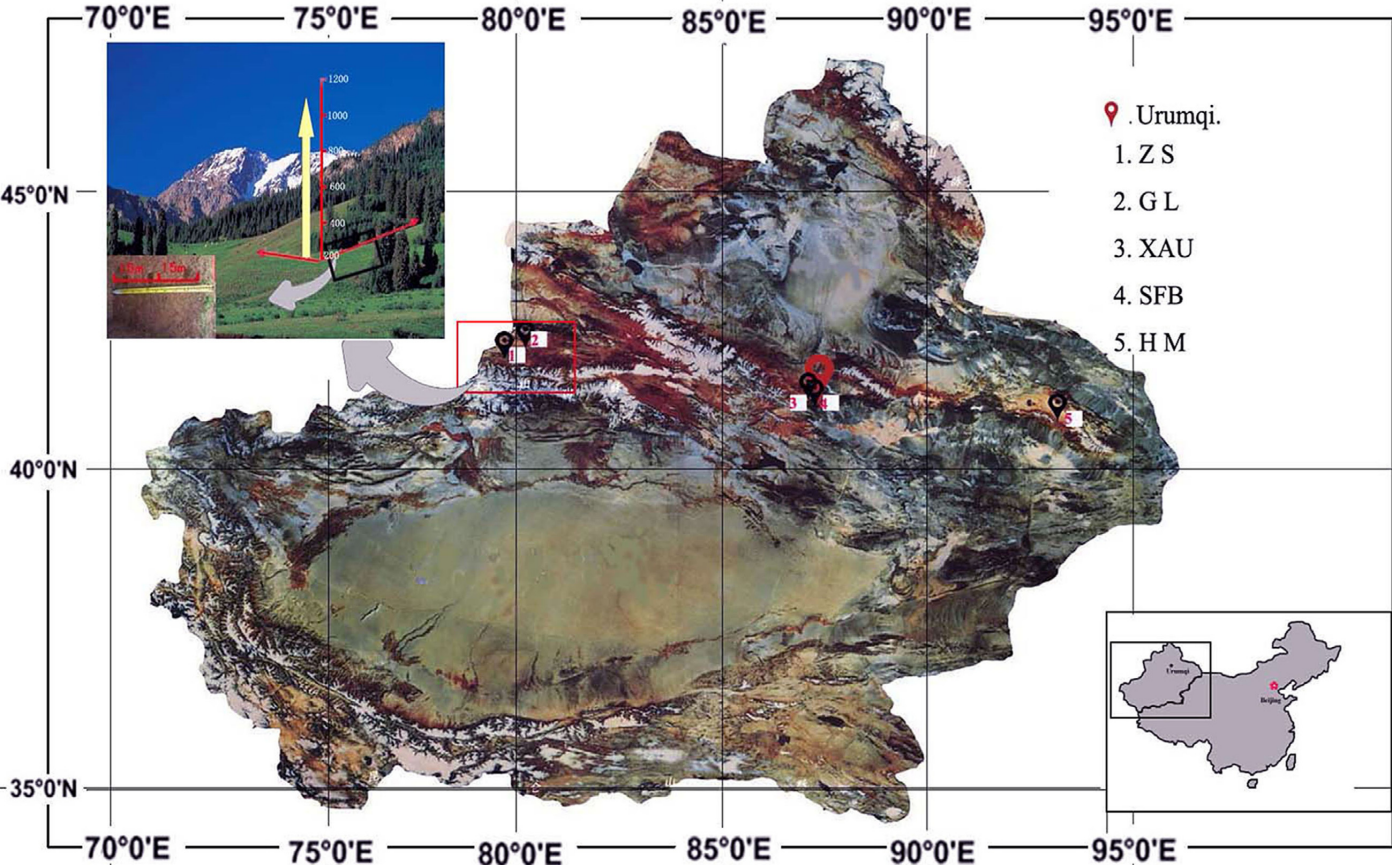
Location of the investigated five sites on the northern slope of Tianshan Mountains, Xinjiang, China.

Five field sites having different climatic conditions in the middle elevations on the northern slops of the Tianshan Mountains, including Zhaosu (ZS) forest center (43°14′N, 81°05′E) and Gongliu (GL) forest center (43°08′N, 82°53′E) in the western Tianshan, Xinjiang Agricultural University (XAU) forest education center (43°16′~43°26′N, 86°46′~86°57′E) and Xingjiang Tianshan forest ecosystem research station of State forestry bureau (SFB) (43°24′~43°26′N, 87°27′~87°28′E) in the central Tianshan, and Hami (HM) forest center (43°18′N, 93°41′E) in the eastern Tianshan (Fig. 1), were selected for observation. A total of sixty‐nine sampling points were scattered on twenty‐three vertical transects determined using a Global Positioning System, and the features (Table 1) were measured according to the previous study (Wang et al. 2006). Real‐time monitoring of soil temperature was carried out during natural regeneration season of *P. schrenkiana* from 2003 to 2012, and the data from each sensor were recorded and transmitted via radio telemetry to a single base station with online data accessing and archiving. The temperatures in the soil surface (3–5 cm) were recorded hourly at each of survey sites during the sampling period, and the temperature data collected from 8:00 am to 8:00 pm were automatically averaged as a daily mean temperature, while the data collected from 8:00 pm to 8:00 am were automatically averaged as night mean temperature.

**Table 1:** Features of the five sites, vertical transects, and sampling points

Sites	Alt. range (m)	No. of transects^a^	No. of points	Soil type^b^	SOM (%)	Total *N* (%)	Density (tree/ha)	DBH (cm)	Height (m)
ZS	2070–2710	4	12	MLGS	25.30	0.83	1134 ± 36	17.9 ± 1.6	11.0 ± 12
GL	1300–2600	7	21	MLGS	24.83	0.81	1158 ± 28	17.7 ± 1.5	13.5 ± 1.2
XAU	1725–2310	4	12	MTGS	23.16	0.76	1364 ± 47	16.4 ± 1.0	11.2 ± 1.2
SFB	1908–2710	5	15	MTGS	23.23	0.76	1728 ± 62	17.1 ± 1.2	12.5 ± 1.2
HM	2230–2810	3	9	MCGS	18.76	0.53	1562 ± 45	17.2 ± 1.2	11.9 ± 0.7

SOM, soil organic matter; DBH, diameter at breast height. Density, DBH, and height are present: mean ± SE.

a In each sites, vertical transects are selected at 200‐m elevation intervals from bottom of range to top. Along each transect, the GPS coordinates three sampling points.

b MLGS: mountain leaching gray‐cinnamon forest soil, MTGS: mountain typical gray‐cinnamon forest soil, MCGS: mountain carbonate gray‐cinnamon forest soil.

### Collection of plant materials

The current‐year needles and cones of *P. Schrenkiana* were collected from the parent trees located at the XAU forest education center (altitude 2198 m, 43°22′58″N, 86°49′33″E) on 28–30 September 2012. All selected *P. Schrenkiana* plants were 30–35 m tall, 80–100 years old, and healthy and had no infection disease. After the collection, the cones were dried in paper bags at room temperature for 7 days and then threshed by hand to get seeds. (The chemical composition of needles and the vigor of seed from various areas show no difference according to our previous study (Li et al. 2009).)

### Extraction and isolation of the active fractions

A total of 200 g of dry *P. Schrenkiana* needles were ground and extracted with distilled water (20 mL/g) at room temperature for 48 h. Sequentially, the solution was extracted three times with the same volume of diethyl ether, ethyl acetate, and *n*‐butanol. The residues were concentrated under the reduced pressure to yield the fractions of diethyl ether (1.96 mg/g dry mass), ethyl acetate (4.67 mg/g dry mass), and *n*‐butanol (14.18 mg/g dry mass), respectively, and the residual water (9.19 mg/g dry mass) after the organic solvent extraction. Bioassay‐guided fractionation of the diethyl ether fraction (3.74 g) with the strongest autotoxicity was conducted in silica gel column chromatography (180 g, silica gel 100–200 mesh, Merck) to give a yellow crystal (986 mg) which was identified as 3,4‐dihydroxy‐acetophenone (DHAP). All the treated solutions were kept in a refrigerator at 4°C for use.

### Bioassay procedure

Stock DHAP solution of 100 mmol/L was prepared by dissolving pure DHAP into the distilled water. Considering the measurable levels of DHAP in the soil, the stock solution was diluted to concentrations of 5, 2.5, 1.0, 0.5, 0.25, and 0.1 mmol/L as treatment solutions and water as control for bioassays. The experiments of seed germination and seedling growth were conducted according to ISTA (1993).

In triplicate, 100 seeds of the surface‐sterilized *P. Schrenkiana* were placed in each sterile Petri dish (15 cm diameters) lined with Whatman #3 filter paper. Each Petri dish was added with 10 mL treatment solution which might be one of the original water extracts, water residue after the organic solvent extraction, the fractions of diethyl ether, ethyl acetate, and *n*‐butanol at 0.1, 0.5, 1.0, and 2.5 mg/mL, and the solutions of DHAP at 5, 2.5, 1.0, 0.5, 0.25, and 0.1 mmol/L. Then, the Petri dishes were placed in programmable illuminated incubator for a light/dark (L/D) cycle of 12 h/12 h accompanied with a temperature cycle of 12/4°C, 14/6°C, and 16/8°C, respectively. Treatments were conducted by a complete randomized design, and each treatment was replicated three times. Germination (radicle emergence) was measured within 7 or 21 days after treatment.

Pregermination of *P. Schrenkiana* seeds were preceded in plastic boxes (20 × 15 × 10 cm) lined with Whatman #3 filter paper for 5–6 days until radicles emerged. A total of 100 grain of successful germination seeds were placed in Petri dishes in three replicates, and 10 mL of various treatment solutions (same as germination) was added to each Petri dish. Seedlings were incubated in an programmable illuminated incubator for a L/D cycle of 12 h/12 h accompanied with a temperature cycle of 14/6°C, 16/8°C, and 18/10°C, respectively. Five seeds were randomly sampled from each Petri dish, and the length of shoot and root was measured with a vernier caliper (GB/T 1214.2‐1996; Measuring Instrument LTD, Shanghai, China). Fresh weight of seedlings was also recorded (Mettler Toledo Instrument Ltd). The measurements were taken on the third day after incubation and continued once every 3 days for a total of 30 days.

### Statistical analysis

The average day and night temperature as well as average month temperature was calculated in Excel software and statistically analyzed linear regression using SPSS software, (SPSS Chicago). All results of seed germination and seedling growth of *P. Schrenkiana* were presented as the mean ± standard error of three replications. The significant differences among treatment solutions and control on seed germination and seedling growth of *P. Schrenkiana* were first examined by ANOVA (*P *<* *0.05) and then analyzed using Fisher's LSD test for multiple comparisons among the different treatments using SPSS software.

## Results

### Change of topsoil temperature at *Picea Schrenkiana* regeneration stage

The changes of soil temperature in the five survey sites showed that mean soil temperatures of day/night in the periods of seed germination from May 15 to June 14 and seedling growth from June 15 to July 15 increased 3.1–3.9 °C/1.1–1.9°C and 1.7–3.3 °C/1.9–2.4 °C, respectively (Table S1). In the germination periods, the minimum soil temperatures of day/night (9.2°C/3.7°C) appeared at the sampling points of ZS and the maximum temperatures (15.6°C/6.5°C) at GL site, respectively. In the seedling growth periods, the maximum soil temperatures during the day (18.3°C) and night (8.8°C) appeared at GL site, and the minimum soil temperatures of 12.3°C during the day appeared at ZS site, while the minimum soil temperatures of 4.9°C during the night appeared at GL site with altitude 2600 m and SFB site with altitude 2700 m.

The data of monthly soil temperature change at the five survey sites have been collected from 2003 to 2012 (Table S1). During the 10 years, average day and night temperatures have increased 0.5–0.6°C and 0.2–0.3°C in the germination period and increased 0.5–0.7°C and 0.3–0.4°C in the seedling growth periods, respectively. Monthly average soil temperature due to anomalous climate change in 2010 was much higher than those in other years. Based on the above analysis, the benchmark day and night temperatures were determined to be 12°C and 4°C for seed germination, and 14°C and 6°C for seeding growth, respectively.

### Effects of water extract and organic fractions on the seed germination with temperature rising

Seed vigor is defined as the sum total of those properties of the seed that determine the level of activity and performance of the seed during germination and seedling emergence. The concept of seed vigor is of vital importance to the seed industry because two seed lots with same germination percentage, but differing vigor, could show significant variation in stand and yield when planted under various stress conditions (ISTA, 1993). Either the germination rate or vigor of *P. Schrenkiana* seeds was measured to determine the effects of water extract or organic fractions as temperature increased (Table 2). In the environment of water alone, the germination vigor of *P. schrenkiana* seeds enhanced significantly as temperature increased. In the presence of water extract (1.25 mg/mL), the temperature rise of 2 to 4°C significantly inhibited the germination vigor and rate of the seeds (*P *< 0.05). For the low‐polar fraction (diethyl ether extraction) at the concentrations of 0.1, 0.5, and 2.5 mg/mL, the initial temperature rise of 2 °C significantly reduced the seed germination vigor and rate, the further temperature rise of 2 °C also enhanced the inhibition but with lesser effect, and at the concentration of 1.0 mg/mL, however, temperature rise of 4 °C showed more significant effect of inhibition to the seed germination (Table 2). For the high‐polar fraction (*n*‐butanol extraction) and medium‐polar fraction (ethyl acetate extraction), a low concentration of 0.1 mg/mL significantly promoted the germination vigor and rate. With increasing temperature by 4 °C, the vigor was significantly enhanced, but not the rate (Table 2). As concentration increased (0.5–2.5 mg/mL), the inhibition of germination vigor and rate was significantly enhanced. On the other hand, temperature rise gave no obvious inhibiting effect on the vigor and rate of seed germination at the concentration over 0.5 mg/mL, but significantly reduced germination vigor and rate at the concentration of 1.0 or 2.5 mg/mL. In addition, the inhibition effect between temperature rise of 2 and 4°C showed no significant difference.

**Table 2:** Effects of water extract and organic fractions on seed germination of *P. schrenkiana* with temperature rising

Germination Vigor (%)/Germination rate (%)	Water	Water extract	Water residue	Diethyl ether fraction (mg/mL)			
				0.10	0.50	1.00	2.50
Temperature (°C)							
12‐4	58 ± 2h/81 ± 2D	37 ± 2m/46 ± 2L	57 ± 3h/80 ± 1D	45 ± 2k/62 ± 2G	24 ± 1r/37 ± 2O	19 ± 1st/22 ± 1TU	12 ± 2uv/12 ± 1W
14‐6	62 ± 1g/82 ± 1CD	34 ± 1no/38 ± 1NO	61 ± 2g/82 ± 2CD	41 ± 1l/57 ± 2H	19 ± 2st/28 ± 1QR	17 ± 1t/20 ± 1U	7 ± 1w/7 ± 1X
16‐8	67 ± 2ef/80 ± 2D	30 ± 2p/32 ± 2P	67 ± 2ef/80 ± 2D	42 ± 2l/56 ± 1HI	19 ± 2st/29 ± 2Q	14 ± 1u/16 ± 2V	6 ± 1w/6 ± 1X

	Ethyl acetate fraction (mg/mL)				n‐Butanol fraction (mg/mL)			
	0.10	0.50	1.00	2.50	0.10	0.50	1.00	2.50
Temperature (°C)								
12‐4	66 ± 2f/84 ± 2B	48 ± 1j/67 ± 2E	32 ± 1op 54 ± 1IJ	18 ± 2st/20 ± 1U	75 ± 2c/86 ± 2AB	52 ± 1i/62 ± 2G	45 ± 1k/56 ± 1HI	27 ± 2q/40 ± 1MN
14‐6	69 ± 1e/82 ± 2CD	47 ± 2jk/65 ± 1EF	31 ± 1p/52 ± 1JK	12 ± 1uv 14 ± 1VW	78 ± 1b/85 ± 2AB	53 ± 2i/63 ± 1FG	38 ± 1m/41 ± 1M	20 ± 1s/26 ± 1RS
16‐8	72 ± 2d/82 ± 1CD	46 ± 2jk/64 ± 2FG	30 ± 1p/51 ± 2K	10 ± 1v/12 ± 1W	82 ± 2a/87 ± 1A	52 ± 2i/64 ± 2FG	36 ± 1mn/40 ± 2MN	18 ± 1st/24 ± 1ST

Data in the table mean average ± SDin three replicates. The same letter next to each average shows that the difference between consistency is not significant, *P* < 0.05.

### Effects of water extract and organic fractions on seedling growth with temperature rising

**Figure 2:**
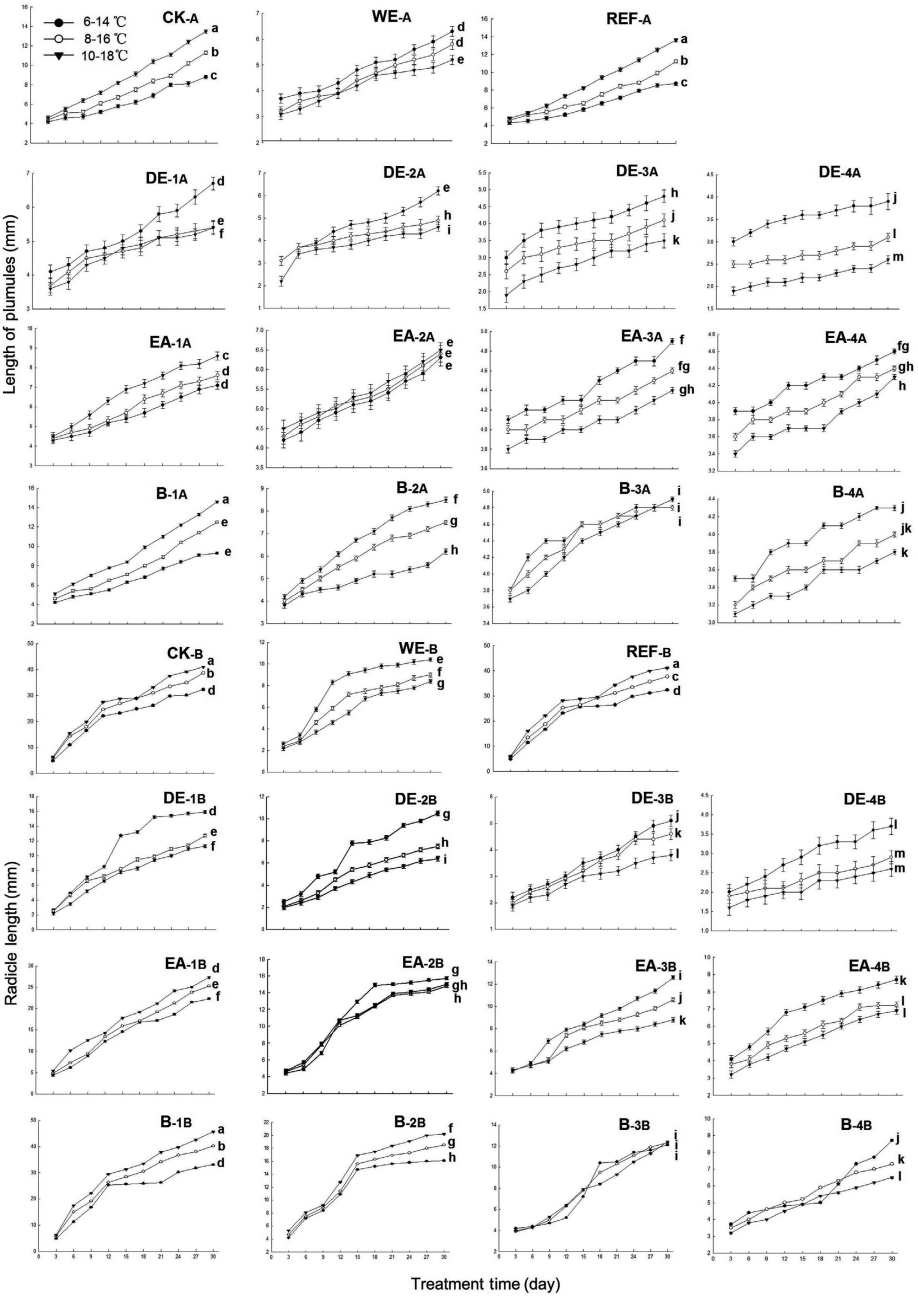
Effects of water extract and organic fractions on radicle and plumules length of seedling of *P. schrenkiana* with temperature rising (CK: water; WE: water extract; REF: residual water after organic solvent fraction; DE: diethyl ether fraction; EA: ethyl acetate fraction; B: butanol fraction; _A_: plumules length; _B_: radicle length; _1,2,3,4_: 0.1, 0.5, 1.0, 2.5 mg/mL, respectively). Line graphs within the same plot followed by the same letter are not different at *P* = 0.05 level according to Fisher's test; each point is the mean of three replicates ± SD.

**Figure 3:**
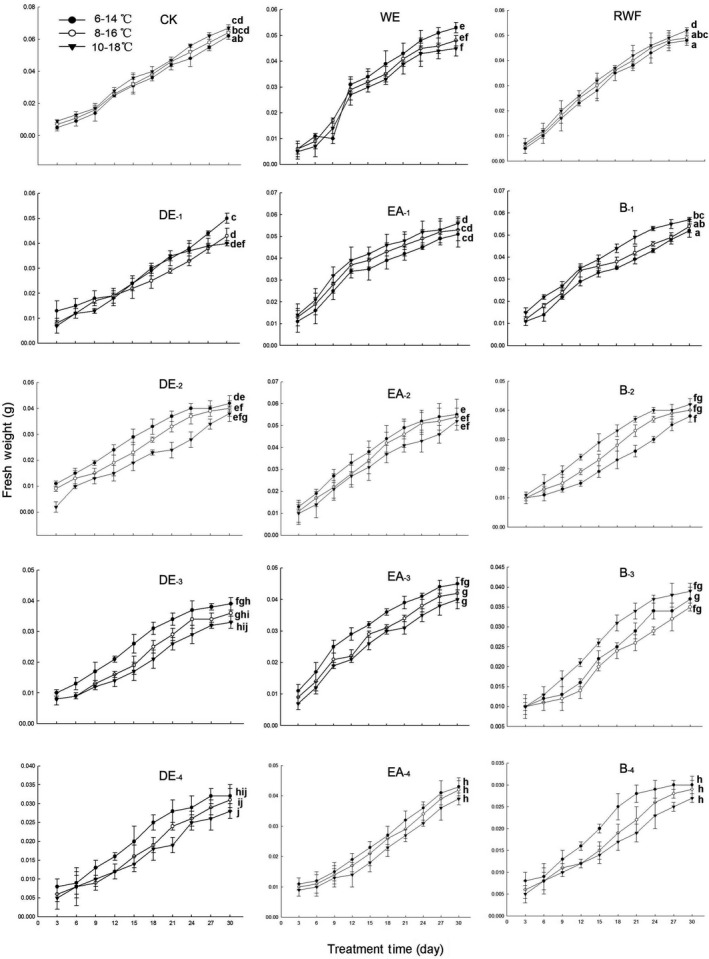
Effects of water extract and organic fractions on fresh weight of seedling of *P. schrenkiana* with temperature rising (CK: water; WE: water extract; RWF: residual water after organic solvent fraction; DE: diethyl ether fraction; EA: ethyl acetate fraction; B: butanol fraction; _1,2,3,4_: 0.1, 0.5, 1.0, 2.5 mg/mL, respectively). Line graphs within the same plot followed by the same letter are not different at *P* = 0.05 level according to Fisher's test; each point is the mean of three replicates ± SD.

The effects of water extract and organic fractions on the seedling growth of *P. schrenkiana* in different temperatures were measured by the plumules length, radicle length, and fresh weight of seedlings (Figs 2 and 3). In the presence of water extract at 1.25 mg/mL, a temperature rise of 2 °C significantly reduced radicle length, but only slightly decreased plumules length and fresh weight of seedling; a temperature rise of 4 °C significantly increased the inhibition of water extract to the seedling growth (*P *<* *0.05). The effect of water residue (1.25 mg/mL) on seed germination and seedling growth did not differ significantly from the effect of water (control).

For the low‐polar fraction, temperature rise of either 2°C or 4°C significantly increased the inhibition to plumules and radicle length of seedling (Fig. 2). Temperature rise of 2°C significantly increased the inhibition on fresh weight at the low concentration of 0.1 mg/mL, but the inhibition effect became less significant with continuously raising temperature or increasing the concentration of this fraction (Fig. 3). For the medium‐polar fraction, temperature rise could significantly increase the plumules and radicle length of seedlings at the fraction concentration of 0.1 mg/mL and significantly reduce the length at the concentration of 2.5 mg/mL. Moreover, the promotion of 0.1 mg/mL fraction on fresh weight significantly increased by the temperature rise of 4 °C, while the inhibition effect of 0.5, 1.0, and 2.5 mg/mL fraction did not decrease by raising temperature (Fig. 3). For the high‐polar fraction, the temperature rise of 4 °C significantly inhibited the plumules length at the concentration of 0.1 mg/mL; the inhibition effect of 1.0 and 2.5 mg/mL fractions was not significantly increased with the temperature rise of 2 °C, but significantly increased with the temperature rise of 4 °C. Moreover, the temperature rise significantly promoted radicle length by the fraction of 0.1 mg/mL, but inhibited the length by the fraction of 0.5–2.5 mg/mL. The inhibition to the fresh weight showed that temperature rise decreased the inhibition of this fraction at 0.1 mg/mL, but did not affect the inhibition of this fraction at 0.5, 1.0, and 2.5 mg/mL (Fig. 3).

### Effects of temperature rising on autotoxicity of DHAP

In general, the key allelochemical DHAP at low concentration of 0.1 mmol/L promoted both the germination vigor and rate, but inhibited at concentrations from 0.25 to 5.0 mmol/L. By comparison, the effect of concentration was dominant, while the effect of temperature rise became less important with increasing concentration. For example, increasing temperature by 4°C remarkably enhanced the promotion effect of 0.1 mmol/L DHAP and significantly increased the inhibition effect of 0.25 or 0.50 mmol/L DHAP, but only slightly increased the inhibition effect of 2.5 or 5.0 mmol/L DHPA on the vigor and rate of seed germination (Table 3).

**Table 3:** Effects of DHAP on seed germination of *P. schrenkiana* with temperature rising

Germination Vigor (%)/Germination rate (%)	DHAP (M)						
	Water	0.10	0.25	0.50	1.00	2.50	5.00
Temperature (°C)							
12‐4	58 ± 1d/81 ± 2CD	64 ± 2c/85 ± 2B	56 ± 2d/79 ± 2D	32 ± 1f/43 ± 2G	19 ± 1h/30 ± 1I	10 ± 1j/18 ± 2L	5 ± 1lm/8 ± 1N
14‐6	62 ± 1c/82 ± 2C	67 ± 1b/87 ± 2AB	51 ± 2e/65 ± 2E	27 ± 1g/38 ± 2H	15 ± 1i/26 ± 1J	9 ± 1jk/17 ± 1L	3 ± 1mn/6 ± 1N
16‐8	67 ± 1b/80 ± 2CD	73 ± 2a/88 ± 2A	49 ± 2e/61 ± 2F	25 ± 1g/38 ± 2H	14 ± 1i/23 ± 2K	7 ± 1kl/14 ± 1M	2 ± 1n/6 ± 1N

Data in the table mean average ± SD in three replicates. The same letter next to each average shows that the difference between consistency is not significant, *P* < 0.05.

**Figure 4:**
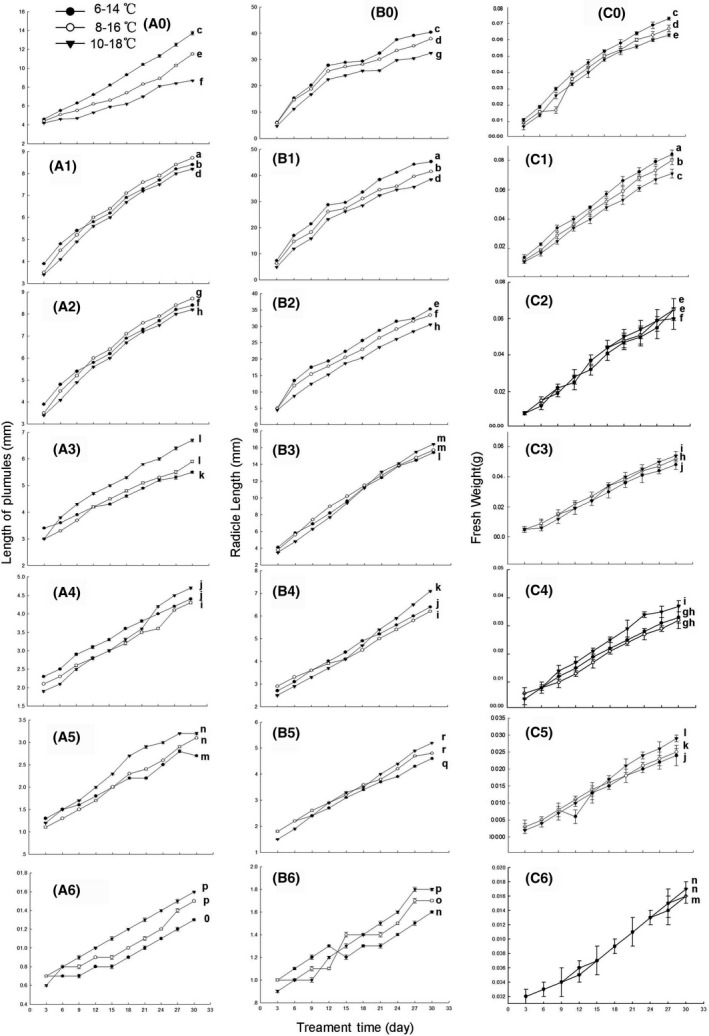
Effects of DHAP on seedling growth of *P. schrenkiana* with temperature rising (A: plumules length; B: radicle length; C: fresh weight of seedling; _0_: water; _1,2,3,4,5,6_: 0.1, 0.25, 0.5, 1.0, 2.5, and 5.0 mmol/L DHAP, respectively). Line graphs within the same plot followed by the same letter are not different at *P* = 0.05 level according to Fisher's test; each point is the mean of three replicates ± SD.

The temperature rise of 2 or 4°C significantly increased the promotion of 0.1 mmol/L DHAP on the plumules length of seedlings, but not affected the inhibition effect of 0.25, 1.0, and 2.5 mmol/L DHAP. The temperature rise of 2°C significantly increased the inhibition of 0.5 and 5 mmol/L DHAP on the growth of seedlings. Also, temperature rising significantly increased the promotion of 0.1 and 0.25 mmol/L DHAP to radicle length of seedlings, did not change the effect of 0.5 and 5.0 mmol/L DHAP, and significantly increased the inhibition of 1.0 and 2.5 mmol/L DHAP. Furthermore, the temperature rise of 4°C significantly increased the promotion of 0.1 mmol/L DHAP and the inhibition of 2.5 mmol/L on the fresh weight of seedlings, did not change the effect of 0.25 and 5.0 mmol/L DHAP, and only slightly increased the inhibition of 0.5 and 1.0 mmol/L DHAP (Fig. 4).

## Discussion

According to the projection of climate warming, forests around the globe will likely undergo major changes of landscape‐scale vegetation in the coming decades. Such changes in forest cover will certainly affect the delivery of important ecosystem services, including biodiversity richness, climate regulation, carbon storage, and water supplies (Foley et al. 2005). Obviously, forests are now under tremendous pressure from global change, and also, forest dynamics becomes one of the greatest sources of uncertainty in predicting future climate (Purves and Pacala, 2008). Interdisciplinary science to integrate knowledge of interacting climate services of forests with the impacts of global change is necessary to identify and understand as yet unexplored feedbacks in the Earth system and the potential of forests to mitigate climate change (Canadell and Raupach 2008). It is hoped that this case study on climate warming to affect the autotoxicity and regeneration of *P. schrenkiana* could enrich and deepen the understandings of the complex interactions between forest evolution and climate change.

Based on the simulation of a regional climate model with 2 × CO_2_, it was predicted that the climate change due to greenhouse effects would increase the mean annual temperature in Northwest China by 2.7°C, with about an increase of 3.0°C in the winter and spring, during the next hundred years (Gao et al. 2003; Shi et al. 2003). Although climate warming has become a global phenomenon, the development trend and impact consequence might be different in various regions and locations (Su et al. 2007). For this reason, analyses are better to be conducted at the local level rather than the large scale. In this work, the field real‐time monitoring showed that soil temperatures of the study sites SFB, ZS, GL, XAU, and HM during the *P. Schrenkiana* germination period (from middle of May to middle of June) changed from 3.41 to 12.62°C, 2.89 to 11.06°C, 3.37 to 12.59°C, 3.68 to 11.75°C, and 3.68 to 12.50°C, respectively, and the temperatures of the five sites during the seedling growth period (middle of June to middle of July) changed from 4.30 to 16.45°C, 3.66 to 13.75°C, 4.23 to 16.56°C, 4.59 to 16.49°C, and 4.96 to 16.50°C, respectively. Also, the average soil temperature at the regeneration stage of *P. Schrenkiana* has increased 0.5–0.7°C at day and 0.2–0.4 °C at night during the last decade. The survey data were very valuable for the study of global climate change and regional responses.

It is well known that various higher plants release a diversity of allelochemicals including phenolics, alkaloids, long‐chain fatty acids, terpenoids, and flavonoids into the environment (Blum 1996). Phenolic mixtures such as vanillic, benzoic, protocatechuic, cinnamic, syringic, and ferulic acids in the litters and rhizosphere soil of *Pinus laricio* have very strong inhibition to its seed germination (Muscolo and Sidari 2006). *p*‐Hydroxybenzoic, gallic, coumaric, ferulic, vanillic, and protocatechuic acids were the allelochemicals responsible for the autotoxicity to the replanted fir trees [*Cunninghamia lanceolata* (Lamb.) Hook] in China (Huang et al. 2003). Understanding of allelopathy in the integrated community and ecosystem context requires to recognize the large number of different processes affected by the same chemical or its derivatives, and the potential for the direct allelochemical effects of plants on each other to be augmented, attenuated, modified, or offset by temperature change. These interactions can enhance or reduce allelochemical production, change the persistence or effectiveness of allelochemicals in soil, and selectively increase or decrease allelochemical concentrations over evolutionary time. Undoubtedly, studying the effect of temperature on plant autotoxicity will help us to recognize the mechanism of allelopathy in bridging the causal relationship between forest evolution and climate warming.

In this preliminary investigation, it was found that there was variable and synergistic effect of temperature, chemical polarity, and concentration on *P. Schrenkiana* regeneration. The inhibition effect of *P. schrenkiana* needles' water extract on the seed germination and seedling growth of *P. Schrenkiana* could be increased with the rising environment temperature. Among the three organic fractions of the water extract, the low‐polar fraction (diethyl ether) showed to be more phytotoxic than other two fractions, causing significant inhibitory effects on the seed germination and growth even at low concentration of 0.1 mg/mL, and the inhibition effect was enhanced with the increasing temperature or concentration. On the other hand, low concentration (0.1 mg/mL) of medium‐polar (ethyl acetate) and high‐polar (*n*‐butanol) fractions could promote the seed germination and seedling growth, and the promotion effect was generally enhanced with increasing temperature; at concentration of 0.25–1.0 mg/mL, however, the two fractions could inhibit the seed germination and seedling growth, and the effects of temperature rise on the inhibition strength were variable; at high concentration of 2.5 mg/mL, the two fractions could result in more significant inhibition to seed germination and seedling growth, and the inhibition strength was significantly enhanced with rising temperature.

It has been found that some compounds could act as plant growth regulators, exhibiting hormesis, or concentration‐dependent stimulatory or inhibitory effects on seedling growth (Batish et al. 2008; Kato‐Noguchi et al. 2010). Weir et al. (2003) discovered that (−)‐catechin isolated from *Centaurea maculosa* stimulated roots growth in *Gaillardia aristata* and *Lobelia erinusat* 10 *μ*g/mL, but had a significant inhibitory effect at 400 *μ*g/mL. Needle‐leached DHAP had a similar effect on some of the plants, which was verified by tests. In this investigation, 0.1 mmol/L DHAP could act as promoter and 0.5 mmol/L DHAP as a inhibitor, and 0.25 mmol/L DHAP might be the inflection point of changing action direction. More interestingly, the concentration of DHAP corresponding to the inflection point decreased as the temperature increased (see the red line in Table 3). In natural forest conditions, one gram of dry soil from a mature *P. Schrenkiana* forest was determined to contain 0.51 ± 0.03 mg DHAP (Ruan et al. 2011). If DHAP in 1 g field soil was dissolved into 15 mL snow or rain water, the concentration of DHAP would be 0.224 mmol/L which was near to the concentration of inflection point. It was hypothesized that the concentration of DHAP inflection point was varied in different plants.

It has been suggested that under field conditions, the self‐circulation mechanisms of *P. schrenkiana* forest ecosystem depended on wildfires to some extent. In other words, the fire seems to be important to maintain the natural regeneration of *P. Schrenkiana* forest ecosystem, probably because long‐term wildfires could suppress and even eliminate the autotoxicity effects of plants. Up to date, policy makers are challenged to categorize all fires as destructive to ecosystems simply because they have long flame lengths and kill most of the trees within the fire boundary. As ecological context matters, however, high‐severity fire regimes are appropriate in some ecosystems, but climate change may modify these fire regimes and ecosystems as well (Stephens et al. 2013). For example, the internal detoxification mechanism of *P. Schrenkiana* forest ecosystem might be artificially interfered and destroyed since the forest fire prevention has been greatly strengthened in the past 80 years. In the result, the effects of autotoxicity on regeneration of *P. Schrenkiana* will become even more prominent. Therefore, understanding the role of plant allelopathy in forest regeneration under the background of global warming is becoming more and more urgent to ecologists and environment managers.

Finally, the controlled studies at our laboratory proved that the leaf leachates of *P. schrenkiana* had autotoxic effects. Accumulation of autotoxins in the rhizosphere was responsible for the scant self‐regression within the established *P. schrenkiana* forests, and the autotoxicity varied with rising temperatures. Similar to most of previous studies, our experiment of testing allelopathy was highly controlled without using soil, which might adsorb compounds and microorganisms to make compounds harmless or use them as a carbon and energy source. In spite of some deficiencies, laboratory bioassays are still valuable to indicate the role of chemical compounds in plant–plant interaction. However, such role should be validated in the field or with natural soils at least. Future research should establish an ecology–mathematical model to quantitatively analyze the influence of allelopathy to forest regeneration under the background of global warming.

## Conflict of Interest

None declared.

## Supporting information


**Table S1**. Linear regression equation on the change of average day and night temperature of soil in stage of *P. Schrenkiana* regeneration at five research sites (2012).
