# Supplementary material for: Effects of climate warming on plant autotoxicity in forest evolution: a case simulation analysis for Picea schrenkiana regeneration
Source: Ecol Evol. 2016 Jul 23;6(16):5854–66. doi: 10.1002/ece3.2315 (PMC4983597; doi:10.1002/ece3.2315)
Supplement: Supplementary file 1 — Table S1. Linear regression equation on the change of average day and night temperature of soil in stage of P. Schrenkiana regeneration at five research sites (2012). [file ECE3-6-5854-s001.docx]

| *Supplementary* Table 1S Linear regression equation on the change of average day and night temperature of soil in stage of *P. Schrenkiana* regeneration at five research sites (2012) | | | | | | | | | |
| --- | --- | --- | --- | --- | --- | --- | --- | --- | --- |
| GL |  | SFB |  | ZS |  | XAU |  | HM |  |
| YD_1_=12.59+0.12x  r=0.57; | Yd_1_=16.56+0.11x  r=0.29; | YD_5_=12.62+0.13x  r=0.54; | Yd_5_=16.45+0.11x  r=0.29; | YD_7_=11.06+0.06x  r=0.25; | Yd_7_=13.75+0.07x  r=0.25; | YD_3_=11.75+0.11x  r=0.58; | Yd_3_=16.49+0.11x  r=0.30; | YD_9_=12.50+0.12x  r=0.57; | Yd_9_=16.51+0.11x  r=0.31; |
| YN_1_=5.23+0.09x  r=0.52; | Yn_1_=5.54+0.19x  r=0.69; | YN_5_=5.26+0.08x  r=0.51; | Yn_5=_5.55+0.19x  r=0.66; | YN_7_=3.02+0.12x  r=0.67; | Yn_7_=4.57+0.16x  r=0.72; | YN_3_=4.56+0.10x  r=0.62; | Yn_3_=5.59+0.19x  r=0.66; | YN_9_=5.11+0.09x  r=0.53; | Yn_9_=5.59+0.19x  r=0.66; |
| YD_2_=11.78+0.11x  r=0.57; | Yd_2_=15.73+0.10x  r=0.31;h2 | YD_7_=12.05+0.15x  r=0.56; | Yd_7_=15.69+0.16x  r=0.32; | YD_9_=11.01+0.06x  r=0.25; | Yd_9_=13.44+0.72x  r=0.29; | YD_5_=11.07+0.14x  r=0.68; | Yd_5_=15.71+0.10x  r=0.32; | YD_11_=11.05+0.14x  r=0.68; | Yd_11_=15.72+0.1x  r=0.30; |
| YN_2_=4.58+0.10x  r=0.61; | Yn_2_=5.40+0.16x  r=0.66; | YN_7_=4.67+0.11x  r=0.58; | Yn_7_=5.38+0.16x  r=0.15; | YN_9_=3.10+0.11x  r=0.64; | Yn_9_=4.28+0.16x  r=0.72; | YN_5_=4.49+0.09x  r=0.60; | Yn_5_=5.41+0.16x  r=0.64; | YN_11_=4.41+0.09x  r=0.61; | Yn_11_=5.38+0.16x  r=0.65; |
| YD_4_=11.10+0.14x  r=0.67; | Yd_4_=14.76+0.14x  r=0.43; | YD_9_=11.02+0.14x  r=0.19; | Yd_9_=14.75+0.14x  r=0.43; | YD_11_=10.71+0.09x  r=0.39; | Yd_11_=13.14+0.10x  r=0.41; | YD_7_=10.77+0.13x  r=0.68; | Yd_7_=14.73+0.14x  r=0.44; | YD_13_=10.04+0.14x  r=0.69; | Yd_13_=14.75+0.13x  r=0.43; |
| YN_4_=4.49+0.09x  r=0.61; | Yn_4_=5.00+0.16x  r=0.66; | YN_9_=4.45+0.9x  r=0.59 | Yn_9_=5.05+0.16x  r=0.64; | YN_11_=2.89+0.13x  r=0.74; | Yn_11_=3.97+0.17x  r=0.75; | YN_7_=4.21+0.09x  r=0.60; | Yn_7_=5.00+0.16x  r=0.64; | YN_13_=3.68+0.08x  r=0.61; | Yn_13_=4.96+0.17x  r=0.65; |
| YD_6_=10.78+0.13x  r=0.68; | Yd_6_=14.41+0.14x  r=0.46; | YD_11_=9.98+0.14x  r=0.68 | Yd_11_=14.40+0.15x  r=0.46; | YD_13_=10.78+0.10x  r=0.35; | Yd_13_=12.82+0.11x  r=0.45; | YD_9_=10.03+0.14x  r=0.68; | Yd_9_=14.01+0.14x  r=0.49; |  |  |
| YN_6_=4.27+0.10x  r=0.64; | Yn_6_=4.98+0.16x  r=0.65; | YN_11_=3.70+0.09x  r=0.61 | Yn_11_=4.69+0.18x  r=0.66; | YN_13_=3.04+0.12x  r=0.67; | Yn_13_=3.66+0.18x  r=0.74; | YN_9_=3.68+0.09x  r=0.61; | Yn_9_=4.59+0.17x  r=0.64; |  | |
| YD_8_=10.41+0.14x  r=0.69; | Yd_8_=14.03+0.14x  r=0.49; | YD_13_=9.81+0.14x  r=0.69 | Yd_13_=12.75+0.15x  r=0.55; |  |  |  |  |  | |
| YN_8_=3.97+0.09x  r=0.61; | Yn_8_=4.61+0.18x  r=0.65; | YN_9_=3.41+0.08x  r=0.60 | Yn_9_=4.3+0.13x  r=0.62; |  |  |  |  | _D_:day from May 15 to June 14;  _N_:night from same time, R-valve (daily);  _d_:day from June 15 to July 14;  _n_: night from same time, R-valve (daily);  _1, 2, 3, 4, 5, 6, 7, 8, 9, 10, 11, 12, 13_ represent the altitude of vertical transects:1400, 1600, 1700, 1800, 1900, 2000, 2100, 2200, 2300, 2400, 2500, 2600, 2700m, respectively.  Data in table mean average ± S.D.in three replicates. | |
| YD_10_=10.08+0.13x  r=0.67 ; | Yd_10_=13.09+0.14x  r=0.49; |  |  |  |  |  |  |  |  |
| YN_10_=3.69+0.09x  r=0.61; | Yn_10_=4.47+0.16x  r=0.64; |  |  |  |  |  |  |  |  |
| YD_12_= 9.86+0.14x  r=0.68 ; | Yd_12_=12.80+0.14x  r=0.53; |  |  |  |  |  |  |  |  |
| YN_12_=3.37+0.09x  r=0.61 ; | Yn_12_=4.23+0.14x  r=0.64 ; |  |  |  |  |  |  |  |  |

| *Supplementary* Table 2S Linear regression equation on the change of average month temperature of soil in stage of *P. Schrenkiana* regeneration at five research sites (2003-2012) | | | | | | | | | |
| --- | --- | --- | --- | --- | --- | --- | --- | --- | --- |
| GL |  | SFB |  | ZS |  | XAU |  | HM |  |
| YD_1_=13.63+0.10x;  r=0.44; | Yd_1_=16.97+0.09x;  r=0.47; | YD_5_=13.44+0.17x  r=0.64; | Yd_5_=16.41+0.02x  r=0.12; | YD_7_=11.15+0.10x;  r=0.56; | Yd_7_=12.99+0.15x;  r=0.54; | YD_3_=13.42+0.16x;  r=0.48; | Yd_3_=16.41+0.11x;  r=0.54; | YD_9_=12.91+0.12x  r=0.58; | Yd_9_=16.13+0.13x  r=0.56; |
| YN_1_=5.84+0.07x;  r=0.59; | Yn_1_=8.06+0.10x;  r=0.47; | YN_5_=4.84+0.8x  r=0.7; | Yn_5=_6.13+0.11x  r=0.49; | YN_7_=3.85+0.47x;  r=0.81; | Yn_7_=6.61+0.10x;  r=0.44; | YN_3_=4.93+0.08x;  r=0.48; | Yn_3_=6.99+0.11x;  r=0.47; | YN_9_=4.80+0.06x  r=0.58; | Yn_9_=6.93+0.09x  r=0.49; |
| YD_2_=12.85+0.12x;  r=0.56; | Yd_2_=15.87+0.09x;  r=0.46; | YD_7_=12.13+0.17x  r=0.63; | Yd_7_=16.89+0.11x  r=0.58; | YD_9_=0.72+0.10x;  r=0.51; | Yd_9_=12.77+0.12x;  r=0.63; | YD_5_=12.57+0.16x  r=0.48; | Yd_5_=15.89+0.10x  r=0.64; | YD_11_=11.80+0.12x  r=0.48; | Yd_11_=15.59+0.08x  r=0.61; |
| YN_2_=5.19+0.08x;  r=0.53; | Yn_2_=7.53+0.08x;  r=0.54; | YN_7_=4.25+0.07x  r=0.62; | Yn_7_=6.56+0.08x  r=0.54; | YN_9_=3.87+0.02x;  r=0.55; | Yn_9_=6.05+0.07x;  r=0.47; | YN_5_=4.44+0.85x  r=0.62; | Yn_5_=6.07+0.11x  r=0.52; | YN_11_=4.18+0.07x  r=0.52; | Yn_11_=6.09+0.06x  r=0.63; |
| YD_4_=11.10+0.14x  r=0.67; | Yd_4_=15.00+0.11x;  r=0.53; | YD_9_=11.58+0.17x  r=0.63; | Yd_9_=17.19+0.04x  r=0.48; | YD_11_=10.21+0.12x  r=0.53; | Yd_11_=12.29+0.11x;  r=0.67; | YD_7_=11.95+0.13x  r=0.49; | Yd_7_=15.68+0.08x  r=0.65; | YD_13_=11.15+0.13x  r=0.60; | Yd_13_=14.67+0.09x  r=0.62; |
| YN_4_=11.89+0.11x;  r=0.56; | Yn_4_=7.08+0.07x;  r=0.57; | YN_9_=3.85+0.09x  r=0.64; | Yn_9_=6.76+0.06x  r=0.49; | YN_11_=3.76+0.03x  r=0.69; | Yn_11_=5.33+0.10x;  r=0.62; | YN_7_=4.29+0.06x  r=0.78; | Yn_7_=5.65+0.08x  r=0.53; | YN_13_=3.77+0.25x  r=0.51; | Yn_13_=5.39+0.06x  r=0.78; |
| YD_6_=5.08+0.05x;  r=0.49; | Yd_6_=14.80+0.09x;  r=0.57; | YD_11_=10.64+0.13x  r=0.45; | Yd_11_=16.93+0.10x  r=0.65; | YD_13_=9.95+0.12x;  r=0.59; | Yd_13_=11.75+0.10x;  r=0.75; | YD_9_=10.63+0.11x  r=0.41; | Yd_9_=15.05+0.07x  r=0.75; |  |  |
| YN_6_=11.29+0.15x;  r=0.65; | Yn_6_=6.91+0.05x;  r=0.56; | YN_11_=3.79+0.07x  r=0.78; | Yn_11_=5.89+0.05x  r=0.83; | YN_13_=3.63+0.03x;  r=0.81; | Yn_13_=4.80+0.07x;  r=0.71; | YN_9_=4.03+0.06x  r=0.74; | Yn_9_=4.98+0.06x  r=0.64; |  | |
| YD_8_=4.58+0.05x;  r=0.63; | Yd_8_=14.33+0.11x;  r=0.65; | YD_13_=10.15+0.13x  r=0.58; | Yd_13_=14.94+0.09x  r=0.62; |  |  |  |  |  | |
| YN_8_=10.81+0.11x;  r=0.67; | Yn_8_=6.01+0.09x;  r=0.71; | YN_9_=3.60+0.07x  r=0.93; | Yn_9_=5.20+0.05x  r=0.80; |  |  |  |  | _D_: average day temperature of seed germination from 2003 to 2012;  _N_: average night temperature of the same time, R-valve (month);  _d_: average day temperature of seeding growth from 2003 to 2012;  _n_: average night temperature of the same time, R-valve (month);  _1, 2, 3, 4, 5, 6, 7, 8, 9, 10, 11, 12, 13_ same to table2.  Data in table mean average ± S.D.in three replicates. | |
| YD_10_=10.69+0.09x  r=0.71 ; | Yd_10_=13.95+0.08x;  r=0.69; |  |  |  |  |  |  |  |  |
| YN_10_=4.03+0.42x  r=0.62; | Yn_10_=5.62+0.08x;  r=0.66; |  |  |  |  |  |  |  |  |
| YD_12_=10.38+0.09x  r=0.77 ; | Yd_12_=12.89+0.09x;  r=0.58; |  |  |  |  |  |  |  |  |
| YN_12_=3.83+0.03x  r=0.67 ; | Yn_12_=4.97+0.06x;  r=0.62 ; |  |  |  |  |  |  |  |  |
